# Construction of a Single Lentiviral Vector Containing Tetracycline-Inducible Alb-uPA for Transduction of uPA Expression in Murine Hepatocytes

**DOI:** 10.1371/journal.pone.0061412

**Published:** 2013-04-23

**Authors:** Jiasi Bai, Jungang Li, Qing Mao

**Affiliations:** Institute of Infectious Diseases, Southwest Hospital, Third Military Medical University, Chongqing, China; George Mason University, United States of America

## Abstract

The SCID-beige/Alb-uPA mouse model is currently the best small animal model available for viral hepatitis infection studies [1]. But the construction procedure is often costly and time-consuming due to logistic and technical difficulties. Thus, the widespread application of these chimeric mice has been hampered [2]. In order to optimize the procedure, we constructed a single lentiviral vector containing modified tetracycline-regulated system to control Alb-uPA gene expression in the cultured hepatocytes. The modified albumin promoter controlled by tetracycline (Tet)-dependent transactivator rtTA2S-M2 was integrated into a lentiviral vector. The full-length uPA cDNA was inserted into another lentiviral vector containing PTight, a modified Tet-responsive promoter. Two vectors were then digested by specific enzymes and ligated by DNA ligase 4. The ligated DNA fragment was inserted into a modified pLKO.1 cloning vector and the final lentiviral vector was then successfully constructed. H2.35 cell, Lewis lung carcinoma, primary kidney, primary hepatic interstitial and CT26 cells were infected with recombinant lentivirus at selected MOI. The expression of uPA induced by DOX was detectable only in the infected H2.35 cells, which was confirmed by real-time PCR and Western blot analysis. Moreover, DOX induced uPA expression on the infected H2.35 cells in a dose-dependent manner. The constructed single lentiviral vector has many biological advantages, including that the interested gene expression under “Tet-on/off” system is controlled by DOX in a dose-depending fashion only in murine liver cells, which provides an advantage for simplifying generation of conditional transgenic animals.

## Introduction

Recently, development of Alb-uPA/SCID mouse model with humanized hepatic cells has become an important approach for studying the mechanism underlying infections by human hepatotropic viruses, like hepatitis B virus (HBV) and hepatitis C virus (HCV) [3]–[5]. The earliest study of substantial repopulation of a mouse liver with human hepatocytes was reported in 2001 by Dandri et al [6] and Mercer et al [7]. In both reports, the liver failure was induced by the expression of urokinase-type plasminogen activator (uPA) that was driven by an albumin promoter. Protein uPA is a strong plasminogen activator which specifically cleaves the plasminogen to form the active plasmin. Plasmin is an enzyme that is able to degrade many proteins, particularly fibrin clot. In this model, uPA/SCID mice, suffering from uPA-induced liver disease, are transplanted early after birth with primary human hepatocytes. These human hepatocytes integrate in the parenchyma and progressively repopulate the diseased mouse liver without losing their normal metabolic functions. Successfully transplanted mice can then be infected with HBV and HCV.

In the early investigations of animal models, an albumin promoter was applied to activate uPA over-expression in the liver. However, irregulated and unspecific overexpression of transgenic product may have unwanted physiological or toxic side-effects. Pharmacological control of gene expression, which can be achieved by using the so-called “on/off” regulatory systems, provides an alternative approach. The ideal controlled over-expression system should permit the investigators to rapidly and reversibly switch on and off transgene expression exclusively in the desired cells or tissue(s) at any time point during development [8]. Reverse tetracycline inducible expression rtTA (Tet-on) system is one of the most prominent and widely-accepted inducible systems so far [9], because (a) the inducer doxycycline (DOX) is well tolerated in animals and has been widely used as an antibiotic; (b) DOX is liposoluble for tissue penetration; (c) DOX can be given orally so to permit rapid gene induction/silencing switch in vivo in a dose-dependent manner [10]; (d) Tet-on system has been studied in the context of numerous viral and non-viral vectors to regulate expression of various genes [11]–[13]; and (e) the level of gene expression in individual cell correlates directly with the dose of inducer, allowing a graded transcriptional response [14]. Currently available Tet-regulated transgenic methods, however, often require the generation of two transgenic strains, one carrying the transgene of interest under the control of the tet operator, and the another the reverse transactivator TA (rtTA) transgene [15]. Crossing these two strains generates progeny with both transgenes thereby allowing regulation of the gene of interest through the administration of tetracycline. Actually, there are too more randomness because the relevant genetic segregation of control elements are frequently lossing or mispairing in the procedure of crossing and screening to gain transgene progeny. Even the yield probability is very low thereby a lot of resources and time has been wasted. Therefore, the generation of a universal tetracycline-regulated expression system within a single cassette provides not only an advantage for simplifying generation of conditional transgenic animals but also a novel strategy for research and development in this field.

In this study, we aimed at: (a) constructing a liver-specific single vector carrying the necessary elements of the regulatory system in a short DNA sequence with minimal leakage and high level of inducible gene expression; (b) using this recombinant lentiviral vector to co-transfect 293T cells for producing infectious lentivirus which then infected mouse hepatic cell line H2.35 cell and various cells from BALB/c mice; and (c) inducing uPA expression by Dox and analyzing its biologic activity in vitro.

## Materials and Methods

### 1. Construction of CN360 and CN361 Lentiviral Plasmid

The coding sequence rtTA2^S^-M2 was excised from the pTet-On Advanced Vector plasmid (SunBio Shanghai, China). A 2.3-kb mouse albumin promoter and enhancer fragment excised from pAlb-EGFP (a kind gift from Prof. Takahiro Ochiya, Japan) was PCR-amplified by primeSTAR HS DNA Polymerase (TaKaRa, Japan) following the operation instructions (Table 1). PCR conditions were 5 minutes at 94°C, 30 cycles of 10 seconds at 98°C, 15 seconds at 55°C and 2.5 minutes at 72°C, and a final 10-minute extension at 72°C. A yielded 2399 bp fragment was cloned into T vector and sequenced. The T vector containing 2399 bp fragment and the pTet-On Advanced Vector plasmid were respectively digested by Cla I and BamH I restriction enzyme (New England Bio Labs, Mass, USA). The resulted fragments were separated by 1.0% agarose gel electrophoresis and retrieved by DNA retrieve KIT (Tiangen, China). The two Cla I and BamH I fragments were conjoined by In-Fusion enzyme (Clontech, USA) at 25°C for 15 minutes and then at 42°C for 15 minutes. The generated plasmid was termed as CN360.

**Table 1:** The primers of constructing newly recombinant lentiviral vector.

Gene		Primer sequence(5′-3′)	Product size	Tm(°C)
albumin	Sense	agatccagtttatcgatctcgagaaccaaccaccggtgcggccgctctagcttccttag	2399 bp	55
	Antisense	tagacatggtggatcccggggttgatagga		
uPA (1)	Sense	atctctagacggtcagcatgggaacaagtg	1414 bp	52
	Antisense	aatggatcctaccatgaaagtctggctggc		
uPA (2)	Sense	cctggagaaggatccgccaccatgaaagtctggctggcg	1340 bp	55
	Antisense	gcgttcgcgatctagatcagaaggccagacctttct		
uPA (3)	Sense	tgaagtttgaggtggagcag	146 bp	55
	Antisense	caggcagatggtctgtatgg		
GAPDH	Sense	acaactttggcattgtggaa	155 bp	55
	Antisense	gatgcagggatgatgttctg		
CN360/N362-A	Sense	TAGACATGGTGGATCCCGGGGTTGATAGGA	323 bp	55
	Antisense	AAATGCTCAAATGGGAGACA		
CN361/CN362	Sense	TAGACATGGTGGATCCCGGGGTTGATAGGA	308 bp	55
	Antisense	GAAGTGATCATAATCAAATATTA		

There primers were used in constructing newly recombinant lentiviral vector. Mouse full length cDNA of uPA was amplified from C57BL/6J mouse kidney by reverse transcription PCR with uPA (1, 2). The expression of target gene uPA was detected with uPA (3) and GAPDH primers. The clones was screened with uppercase primers.

The coding sequence pTigh was excised from the pLVX-Tight-Puro Vector plasmid (SunBio, Shanghai China). Mouse full length cDNA of uPA was amplified from C57BL/6J mouse kidney by reverse transcription PCR (Table 1). PCR conditions and sequence confirmation were identical as abovementioned. The mouse uPA fragment was then amplified by primeSTAR HS DNA Polymerase (TaKaRa, Japan) (Table 1). PCR conditions were 5 minutes at 94°C, 30 cycles of 30 seconds at 95°C, 30 seconds at 55°C and 30 seconds at 68°C, and a final 10-minute extension at 72°C. The yielded 1340 bp fragment and the pLVX-Tight-Puro Vector were respectively digested by Xba I and BamH I restriction enzyme, and subjected to a 1.0% agarose gel electrophoresis for separation. The two separated Xba I and BamH I fragments were retrieved by DNA retrieve KIT (Tiangen, China), and were conjoined by In-Fusion enzyme at 25°C for 15 minutes and then at 42°C for 15 minutes. The generated plasmid was termed as CN361.

### 2. Construction of CN362-A and CN362 Lentiviral Plasmid

The lentivial vector CN360 and the slightly modified lentiviral plasmid pLKO.1 (CN125, data not shown) were respectively digested by Age I and Avr II restriction enzymes, and the resulted fragments were subjected to 1.0% agarose gel electrophoresis for separation. The two separated Age I and Avr II fragments were retrieved by DNA retrieve KIT (Tiangen, China), and then conjoined by T4 Ligase enzyme at 22°C for 30 minutes. The generated plasmid was termed as CN362-A. The lentivial vector CN361 and The lentivial vector CN362-A were respectively digested by Age I and Xho I restriction enzyme, and subjected to 1.0% agarose gel electrophoresis for separation. The two separated Age I and Xho I fragments were retrieved by DNA retrieve KIT (Tiangen, China), and then conjoined by T4 Ligase enzyme at 22°C for 30 minutes. The generated plasmid was termed as CN362.

### 3. Co-transfection of Recombinant Lentivirus Vectors and CN362 Lentivial Vector into 293T Cells

Recombinant lentiviruses were produced by transfecting 293T cells with the lentiviral expression plasmid CN362 and the packaging plasmids that are psPAX2 of gag/pol and pMD2.G of VSV-G using Frans-EZ (SunBio, Shanghai) reagent. 293T cells (6×10^5^) were cultured in a 10-cm tissue culture plate with opti-MEM (GIBCO, USA). Transfection was performed when the cell density reached 30%–40% confluency. Solution A was prepared by adding 0.5 ml (0.5 mg/ml) CN362 plasmid, 1 ml (0.2 mg/ml) PMD2.G and 0.5 ml (0.2 mg/ml) psPAX2 plasmids (diluted by opti-MEM medium), and then Opti-MEM medium to 18 ml in a 50-ml tube. Solution B was prepared by adding 0.5 ml Frans-EZ in another 50-ml tube and then Opti-MEM medium to 18 ml. Transfection solution was prepared by slowly adding solution B to solution A. The mixture was agitated and then left in the hood at room temperature for 20 min. Three ml of the prepared transfection mixture was added to a plate of 293T cells and the cells were cultured routinely. After 6 hours of culture, the culture medium was exchanged with DMEM (GIBCO, USA). Infectious lentiviruses were harvested at 48 hours post-transfection and then concentrated. The infectious titer was determined by real-time quantitative PCR to determine WPRE-tagged positive rate in 293T cells.

### 4. Infection of H2.35, Lewis Lung carcinoma, Primary Kidney, Primary Hepatic Interstitial and CT26 Cells with Lentivirus

All animal studies were reviewed and proved by the Institutional Review Board of the Southwest Hospital, Third Military Medical University (Chongqing, China) for care and maintenance of laboratory animals. Kidney and hepatic interstitial tissues were always obtained from animals deeply anesthetized with intraperitoneal injections. Animals were terminated with anaesthetic overdose before removal of kidney and hepatic interstitial tissues. All efforts were made to minimize suffering. The primary culture was established initially in Dulbecco's modified Eagle's medium (DMEM) supplemented with 15% fetal bovine serum (FBS) and was maintained in DMEM supplemented with 10% FBS. H2.35 (ATCC Number: CRL-1995™), CT26 (ATCC Number: CRL-2639™) and Lewis lung carcinoma Cells were purchased from BXGK Tech Dev Co, Ltd. (Beijing, China).

H2.35, Lewis lung carcinoma, primary kidney, primary hepatic interstitial and CT26 cells were cultured at a density of 2×10^5^ cells per well in 6-well tissue culture plates with DMEM (low glucose) containing 4% FBS and 200 nM dexamethasone at 33°C in an atmosphere of 10% CO_2_. All primary cells came from BALB/c mice. After 24 hours, the cells were infected with our newly combinant lentiviuses, positive control and negative control. 5 µg/ml polybrene was added in each well. The positive control was the modified pLK0.1 vector without uPA-Alb-rtTA fragment but with fluorescence labeling. We infected the H2.35 cells and other various cells with different titer of concentrated positive control lentivial vectors respectively and chosen the titer which can infect more than 80% of the H2.35 cells and other various cells as the most suit infected concentration and the multiplicity of infection(MOI) of positive control is 10. Then, we used this MOI for our newly recombinant lentivirus to infect H2.35 cells and various cells from mice, and detected the infection efficiency as the integration units of 10^5^∼10^6^ per ml by using quantitative RT-PCR to detect WPRE fragment. After 12 hours of infection, H2.35 cells were exchanged with 1 ml per well of fresh culture medium containing various concentrations of DOX (0, 1, 3, 5, 7 and 10 µg). All cells were exchanged with 1 ml per well of fresh culture medium containing 10 µg DOX for additional 48 hours at 37°C in an atmosphere of 5% CO_2_.

### 5. Real-time Quantitative PCR (RT-qPCR)

After induction with various concentrations of DOX for 48 hours, total RNA was extracted from the cells with Trizol according to the manufacturer's instructions. DNase I was used to treat the total RNA extraction following the protocols. Reverse transcription was performed with a PrimerScript RT reagent Kit (TaKaRa, Japan) following the instructions. The yielded cDNA was used for PCR reactions that include 2 µl of each cDNA dilution and 7.2 µl of water in a total of 20 µl solution containing 10 µl of SYBR Green Real-time PCR Master Mix, and 0.2 µmol/L of sense and antisense primers. Serially diluted plasmid containing WPRE cDNA was utilized to construct a standard curve. The cutoff point (Ct) of each sample was plotted on the standard curve and the mRNA copy numbers were calculated. The GAPDH gene was used as an endogenous control. The relative mRNA levels were expressed as a ratio of uPA to GAPDH.

### 6. Western Blot Analysis

After induced with DOX for 48 hours, the cells were lysed using M-PER (Thermo Pierce, USA) lysis buffer. Protein was extracted and heated at 100°C for 10 minutes. Fifteen µl of each sample and 5 µl of Plus marker (MBI) were loaded into 10% polyacrylamide gel. Samples were electrophoresed at 120 V for 90 minutes and then transferred to PVDF membranes at 100 V, 115 mA for 80 minutes using a wet-dry transfer apparatus (Bio-Red, USA). Membranes were blocked in 5% non-fat dry milk for 2 hours at room temperature and incubated with uPA primary antibodies (Santa Cruz biotechnology, USA) overnight at 4°C. The membranes were then washed with T-PBS containing 0.05% Tween-20 followed by 2-hours incubation with a goat anti-mouse secondary HRP-conjugated antibody. After final washing with T-PBS, the membranes were developed by using chemiluminescence to detect the protein of interest.

## Results

### 1. Construction of Tet-Regulated Lentiviral Vector

**Figure 1:**
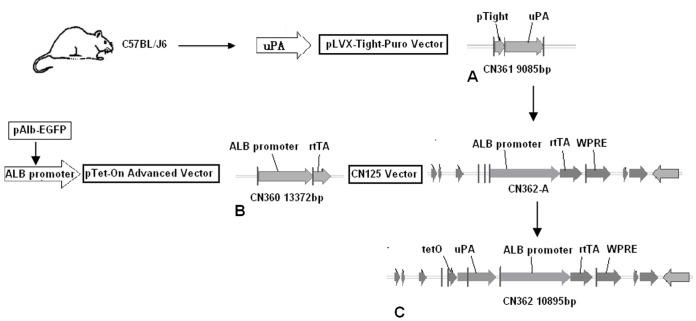
Schematic illustration of the experimental procedure of recombinate lentiviral vector CN362. (**A**) Albumin promoter fragment from pAlb-EGFP plasmid was inserted in the pTet-On Advanced Vector plasmid, and termed CN360. (**B**) uPA fragment from the kiney of C57BL/J6 mouse was inserted in the pLVX-Tight-Puro Vector plasmid, and termed CN361. (**C**) Albumin promoter and rtTA fragments from CN360 interchange into CN125 plasmid, and termed CN362A. uPA and teto fragments from CN361 were inserted in CN362A. and termed CN362.

To establish a DOX-dependent gene expression system with lentiviral vector, we inserted the expression cassettes into two lentiviral vectors respectively (Figure 1); the uPA fragment cloned from the kidney of C57BL/J6 mouse was inserted into the downstream of pTight in the pLVX-Tight-Puro “CN361” (Figure 1A) while the albumin promoter and enhancer were inserted into the upstream of rtTA-Advanced (rtTA2S-M2) in pTet-On Advanced “CN360” (Figure 1B). The vectors were then ingested by Age I and Avr II restriction enzymes and recombined in the modified vector of pLKO.1 cloning vector (termed CN125 lentiviral vector plasmid) to successfully construct a single lentiviral vector termed as plasmid CN362 (Figure 1C). Because CN125 lentiviral vector plasmid contains multiple cloning sites (MCS: Sbf I, Nhe I, BstX I, Pml I, BamH I, Xma I, Not I, Xba I and Avr II), it allows us to insert transgenes under the same bidirectional promoter upstream of the WPRE-included fragment, and delete the DNA cassettes of CMV, puroR and EF1a promoter. The resulting CN360 and CN361 lentiviral vectors were used to generate first generation lentiviral CN362 vector by recombination with CN125 lentiviral backbone vector (data not shown). To obtain liver-targeted gene expression, albumin promoter was selected to control rtTA expression in transfected mouse H2.35 cells in vitro. We also employed the recently described synthetic rtTA2S-M2 simultaneously for tight regulation and stable expression of the Tet-on system. In our single vector, we still maintained some useful functional fragments of woodchuck hepatitis virus posttranscriptional regulatory element (WPRE), a Rev-response element (RRE), and a central polypurine tract (cPPT) element.

### 2. Construction of Single Lentiviral Vector for Liver-Specific Regulable Gene Expression

**Figure 2:**
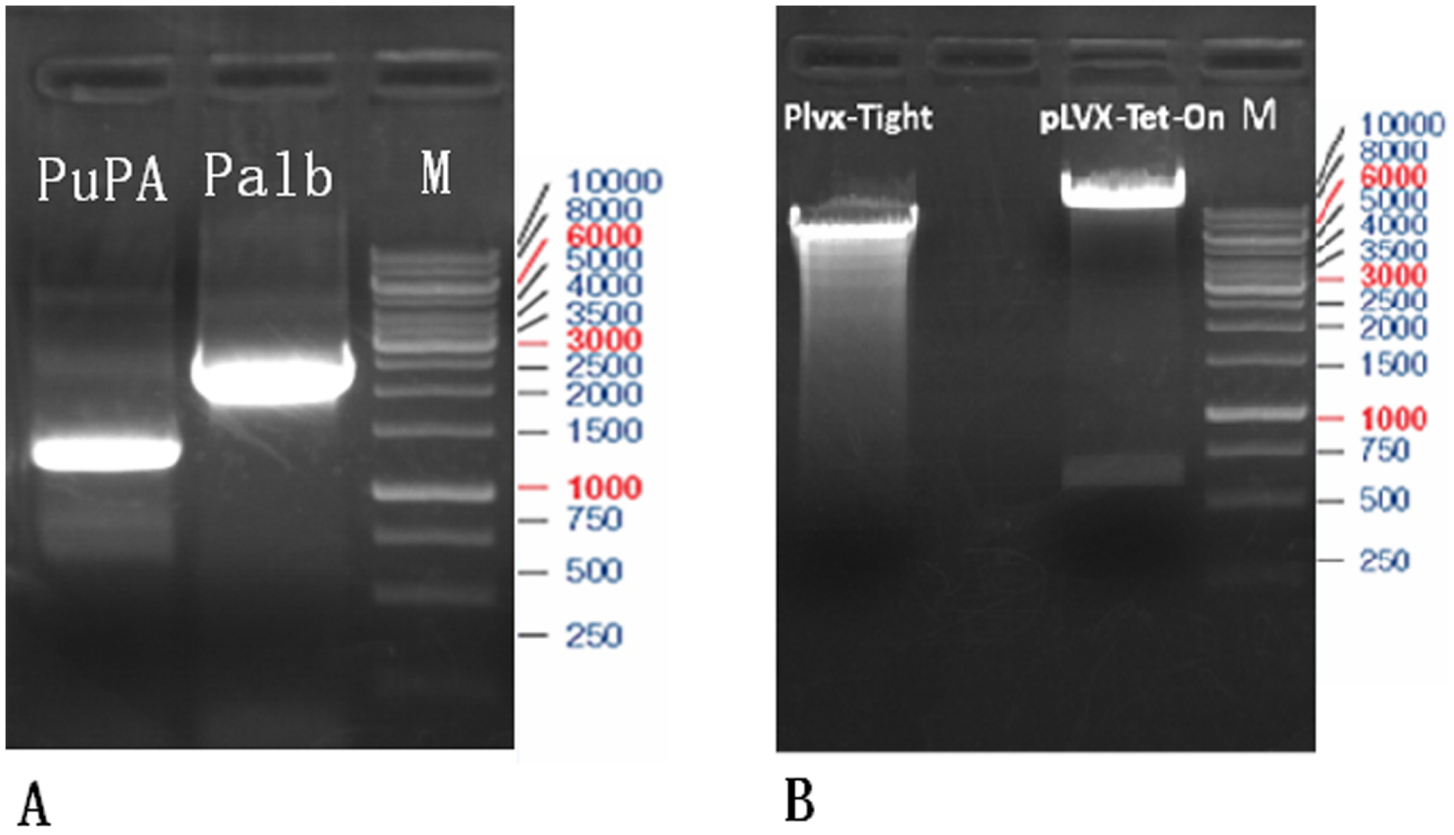
Purification and analysis of PCR products. (**A**) Palb and PuPA fragments were amplified by PCR and run on 1.0% agarose gel. Lane M, DNA ladder. (**B**) pLVX-Tet-on Adanced Vector was digested by Cla I and BamH I restriction enzyme (New England, BioLabs), and pLVX-Tight-Puro Vector was digested by Xba I and BamH I restriction enzyme. The yielded products were run on 1.0% agarose gel. Lane M, DNA marker.

**Figure 3:**
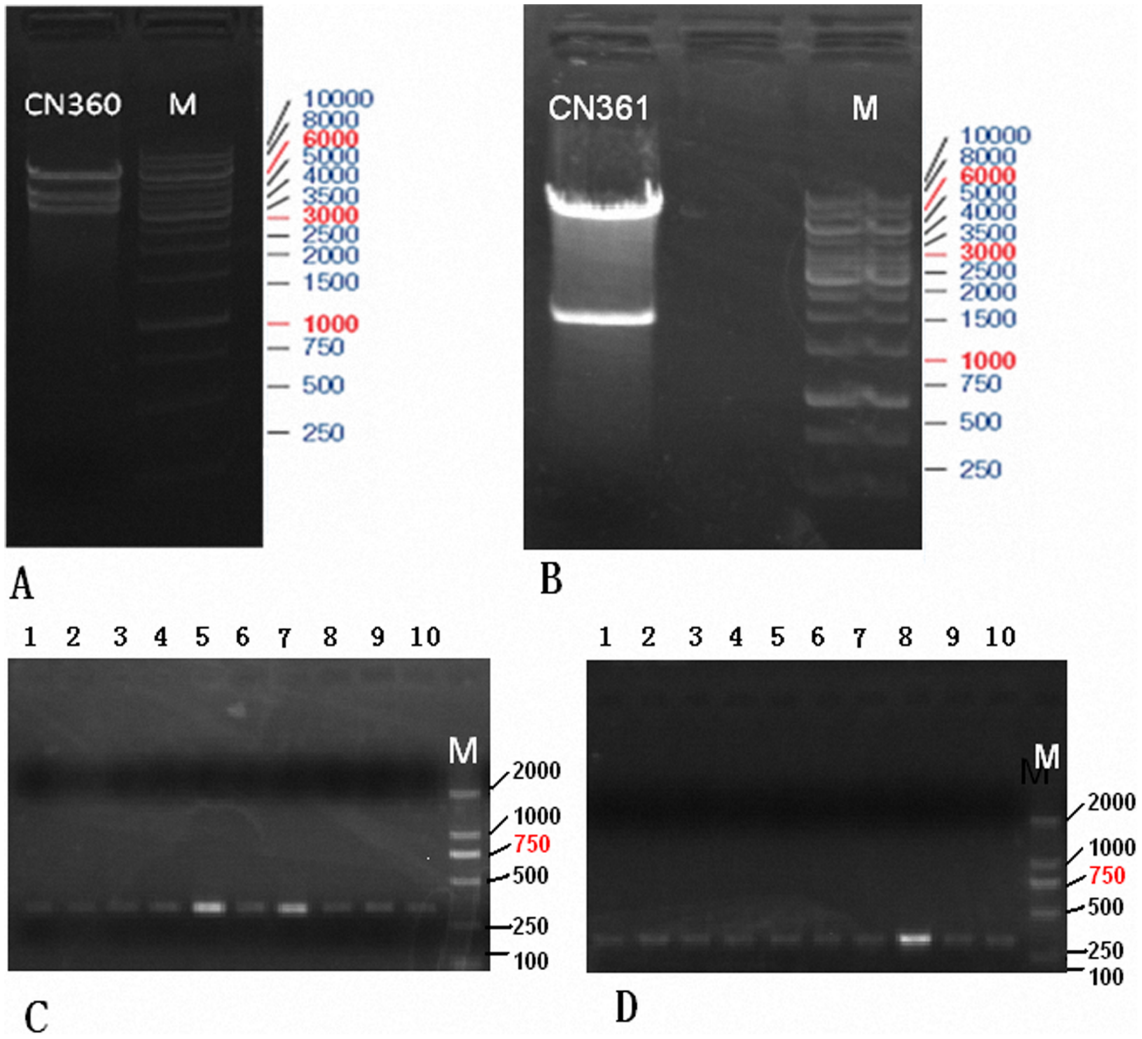
Purification and analysis of PCR products on 1.0% agarose gel electrophoresis. (**A**) CN360 Vector was digested by Age I and Avr II restriction enzyme (New England,BioLabs) and run on 1.0% agarose gel, and the 3270 bp fragment was retrieved. Lane M, DNA ladder. (**B**) CN361 Vector was digested by Age I and Xho I restriction enzyme (New England,BioLabs) and run on 1.0% agarose gel, and the 1780 bp fragment was retrieved. Lane M, DNA ladder. (**C**) Semi-quantitative RT-PCR of positive screened recombinant clones. The producing fragment was 323 bp (Lane 5and 7) and termed CN360. Lane M, DNA ladder. (**D**) Semi-quantitative RT-PCR of positive screened recombnant clones. The producing fragment was 308 bp (Lane 8) and termed CN361. Lane M, DNA ladder.

There are two basic variants of the tetracycline-inducible expression system: the tTA (Tet-off) system [16] and the rtTA (Tet-on) system [17]. Usually, if a gene is to be kept inactive for most of the time and turned on only occasionally, Tet-on system appears to be more appropriate than Tet-off system. Moreover, of these two systems, rtTA system is more suitable for rapid induction of gene expression. But unfortunately, leaky expression, which is derived from both the inherent defects in Tet-based systems and the promoter leakiness caused by promoter-dependent or integration site-dependent effects, compromises the desired stringent regulation of transgene expression [18]–[20]. Thus, we chose to use its newest version, rtTA2s-M2 [21]. To avoid non-specific expression of our interested gene “uPA” (Figure 2A) in other mouse organs in addition to liver, we selected liver-specific albumin promoter (Figure 2A) consisting of a 330 bp long mouse albumin proximal promoter and 2100 bp long enhancer to regulate uPA expression. First, we inserted the fragment of albumin promoter in the upstream of rtTA2s-M2 in the pTet-On Advanced Vector plasmid (Figure 2B) and yielded the lentiviral vector “CN360” (Figure 3A). Clone was screened of the 323 bp fragment (Figure 3C). The orientation of cloned insert and sequence were confirmed by DNA sequencing. We then inserted the fragment of interest gene “uPA” into the downstream of pTight in the pLVX-Tight-Puro Vector (Figure 2B) and yielded another lentiviral vector “361” (Figure 3B). Clone was screened of the 308 bp fragmant (Figure 3D). The orientation of cloned insert and sequence were confirmed by DNA sequencing.

**Figure 4:**
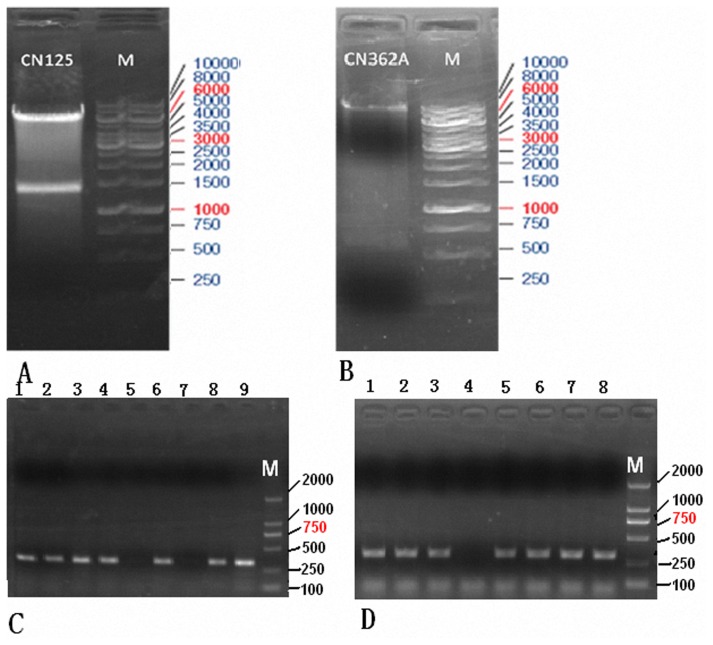
Purification and analysis of PCR products on 1.0% agarose gel electrophoresis. (**A**) CN125 Vector was digested by Age I and Avr II restriction enzyme (New England, Biolabs) and run on 1.0% agarose gel. The 6400 bp fragment from CN125 Vector was retrieved. Lane M, DNA ladder. (**B**) CN362-A Vector was digested by Age I and Avr II restriction enzyme (New England, Biolabs) and run on 1.0% agarose gel. The 9120 bp fragment from CN362-A was retrieved. Lane M, DNA ladder. (**C**) Semi-quantitative RT-PCR of positive screened recombinant clones. The product was 323 bp fragment (Lane 1,2,3,4,6,8 and 9) and termed CN362-A. Lane M, DNA ladder. (**D**) Semi-quantitative RT-PCR of positive screened recombnant clones. The product was 308 bp fragment (Lane 1,2,3,5,6,7 and 8) and termed CN362. Lane M, DNA ladder.

Previously, procedures to obtain tetracycline-regulated transgenic strains need to generate two transgenic strains first: one carrying the transgene of interest under the control of the tet operator, and the another the reverse transactivator TA (rtTA) transgene. Crossing two lines generates progeny with both transgenes, which allows regulation of the gene of interest through the administration of tetracycline. However, crossing and analysis of animals transgenic for each individual component of the system is costly and time consuming. Genetic segregation of control elements during breeding may also be a problem. Therefore, the two fragments in CN360 and CN361 lentiviral vectors were digested by restriction enzymes respectively, and one of them derived from CN360 was inserted into the MCS site of “CN125” (Figure 4A) to yield CN362-A (Figure 4B). Clone was screened of the 323 bp fragmant (Figure 4C), and the orientation of cloned insert and sequence were confirmed by DNA sequencing. Eventually, the destined lentiviral vector CN362 was constructed while another fragment derived from CN361 was inserted into the MCS of CN125 lentiviral vector in identical orientation. The clone was screened of the 308 bp fragment (Figure 4D), and the orientation of cloned insert and sequence were confirmed by DNA sequencing.

### 3. Regulation of uPA and DOX Dose-dependence Study in Vitro

**Figure 5:**
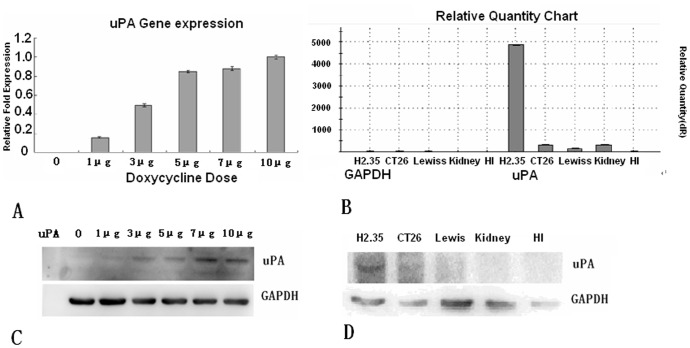
The biological functions of recombined lentiviral vector were verified by RT-PCR and Western blot in target cells. (**A**) The expression of uPA mRNA augmentation following the Dox dose increasing. In 10 µg of DOX dose, the expression of uPA mRNA augmentation to pinnacle; and in 5 µg, the expression of uPA mRNA increased to the platform. (**B**) The expression of uPA mRNA augmentation only in H2.35 cells. (**C**) The augmentation of uPA protein abundance following the increase of inducer Dox dose. (**D**) The abundance of uPA protein was detected by Western blot analysis only in H2.35 cells.

Recombinant lentiviral particles were assembled in the supernatant by transfection of 293T cells with lentiviral expression plasmid CN362, packaging plasmid psPAX2 containing gag/pol and envelope plasmid pMD2.G containing VSV-G. We infected H2.35 mouse hepatic cells with recombinant lentivirus. After incubation for 12 hours, we changed the medium with fresh culture medium containing various concentrations of the inducer DOX (0, 1, 3, 5, 7 and 10 µg) and continued culture for additional 48 hours. Quantitative RT-PCR was employed to detect the expression of uPA mRNA. As shown in Figure 5A, the expression of uPA mRNA was augmented as DOX dosages increased. DOX at 1 µg began to augment the expression of uPA mRNA. When DOX reached to 5 µg, the augmentation of uPA mRNA expression reached to platform and 10 µg to pinnacle. Western blot analysis also detected the DOX-induced increase of uPA protein in the H2.35 cells infected with recombinant lentivirus in a dose-dependent fashion (Figure 5C). Moreover, neither uPA mRNA expression nor uPA protein was detected in the absence of inductor. These results indicate that CN362 satisfies our initial desire for a single lentiviral vector.

It has been reported in the previous studies that neonatal hemorrhaging was the cause of neogenesis mice death and infertility in SCID-beige/Alb-uPA mouse [22]–[23]. Such side-effect can be eliminated through spatiotemporal control of overexpression of the target gene exclusively in the liver. We carried out infection of our recombinant lentivirus with H2.35 cell, Lewis lung carcinoma cell, primary kidney cell, primary hepatic Interstitial cells and CT26 cell derived from mice respectively. After 12 hours, the cultures were changed with fresh medium that contained 10 µg of DOX and incubated for additional 48 hours. Quantitative RT-PCR and western blot were respectively employed to detect the expression of uPA mRNA and protein. As shown in Figure 5B and Figure 5D, the augmentation of uPA mRNA and protein was only detected in H2.35 cells. Therefore, the recombinant lentiviral vector satisfy our desire of which uPA is exclusively augmented in the liver cells.

## Discussion

To explore a novel strategy for establishing transgenic animal model, we constructed a single lentiviral vector and confirmed its biological functions in vitro. Lentiviral vector is being used as a chosen candidate for its capability for stable integration and long-term expression of the transgene. Lentiviral vector is also a favorable vector for biological research and gene therapy trials because of their ability to infect both dividing and non-dividing cells. In our study, the recombinant lentivirus infected H2.35 cells with a lower 10^5^∼10^6^ integration units per ml, and gained higher expression of interested genes. When inducer DOX was 5 µg, the expression of uPA mRNA was 256 times to control; when inducer DOX was 10 µg, the expression of uPA mRNA was 512 times.

A variety of methods for integrating the tetracycline-inducible expression components into a single vector have been described [24]–[26]. Upon designing and constructing the single lentiviral vector, we employed the tetracycline-regulated expression system that is being the most reliable one of several inducible expression systems for mammalian cells. Our constructed vector will also be ready for subsequent replacement of tetracycline-induced gene expression cassette of interest based on the choice of viral and non-viral delivery backbones. We inserted the expression cassettes into two final destination vectors, the uPA fragment was inserted into the downstream of pTight in the pLVX-Tight-Puro “CN360” and the fragments of albumin promoter and enhancer were inserted into the upstream of rtTA-Advanced (rtTA2S-M2) in pTet-On Advanced “CN361” respectively. The “CN361” was then digested by Age I and Avr II restriction enzyme and recombined in the modified vector of pLKO.1 cloning vector to successfully construct a single lentiviral vector, “CN362”. In our experiments, combining the pTigh and rtTA-Advanced (rtTA2S-M2) together have constructed an integrity of regulated expression system, which allowed us to easily replace or insert the genes of interest in the sites and will be useful for other researcher to facilitate cloning a variety of domains.

Our construction strategy took advantage of rtTA-Advanced (rtTA2S-M2), an improved version that contains the reverse Tet-controlled transactivator protein (rtTA) and a modified Tet-responsive promoter, pTigh. rtTA-Advanced (rtTA2S-M2) is more sensitive to DOX and yields lower background expression than the original rtTA used in the Tet-on System [27]. It is fully synthetic, lacks cryptic splice sites, and is codon-optimized for stable expression in mammalian cells. In our experiments, 1 µg/ml DOX was able to induce the uPA mRNA expressing which reached to platform at 7 µg/ml and 10 µg/ml DOX in H2.35 cells. In parallel to uPA mRNA, uPA protein was also induced in H2.35 cells.

pTigh has its own characteristics of a modified minimal CMV promoter, and seven direct repeats of a 36 bp regulatory sequence that contains the 19 bp tet operator sequence [28]. In our constructed vector, it still remained some useful functional fragments of the woodchuck hepatitis virus posttranscriptional regulatory element (WPRE) to promote RNA processing events and enhance nuclear export of viral and transgene RNA [29], leading to increased viral titers from packaging cells, and enhanced expression of our gene of interest in the target cells. In addition, the vector includes a Rev-response element (RRE) to further increases viral titers by enhancing the transport of unspliced viral RNA out of the nucleus [30]. Finally, our constructed vector contains a central polypurine tract (cPPT) element to increase nuclear importation of the viral genome during target cell infection, resulting in improved vector integration and more efficient transduction [31].

Although a modified minimal CMV promoter could drive DOX-dependent expression of our construct in H2.35 cells, it was insufficient to drive high expression of uPA mRNA (date not shown). Thus, we inserted the albumen promoter and enhancer into the upstream of tetracycline controlled transactivator, which had two significant functions of driving uPA mRNA expressing and limiting uPA mRNA expressing in mouse hepatocytes. When the recombinant lentivirus infected the cells from differentiated mouse cells, we found that the expression of uPA mRNA and uPA protein only increased in the H2.35 cells but not other cells. Thus, we believe that the modularly-designed single-vector tet system presented here would provide an ideal system for the tissue specific expression of tTA or rtTA as well as the controlled expression of a transgene from the tetracycline response element. This system could be used for a variety of genetic studies where a single cassette is advantageous.
